# Changing epidemiology of the tick-borne bovine parasite, *Babesia divergens*

**DOI:** 10.1186/1756-3305-7-S1-O8

**Published:** 2014-04-01

**Authors:** A Zintl, G McGrath, L O'Grady, J Fanning, K Downing, D Roche, M Casey, JS Gray

**Affiliations:** 1 School of Veterinary Medicine, University College Dublin, Ireland; 2 Central Veterinary Laboratory, Department Agriculture, Food & the Marine, Dublin, Ireland; 3 Irish Cattle Breeding Federation, Bandon, Co Cork, Ireland; 4 Growth from Knowledge, GfK-Ireland, Market Research Company, Ireland; 5 School of Biology and Environmental Science (Emeritus Professor), University College Dublin, Ireland

## 

Bovine babesiosis is caused by the tick-borne blood parasite, *Babesia divergens*. A survey of veterinary practitioners and farmers in Ireland in the 1980's revealed an annual incidence of 1.7% associated with considerable economic losses. However, two subsequent surveys in the 1990's indicated a decline in clinical babesiosis.

Recent evidence from continental Europe suggests that, probably due to climate change, the distribution of the tick vector of *B. divergens*, *Ixodes ricinus *is extending to more northerly regions and higher altitudes. In addition, milder winters are thought to increase the window of tick activity.

In order to determine whether any such changes have affected the incidence of bovine babesiosis in Ireland, a questionnaire survey of farmers and veterinarians was carried out and compared against data from previous surveys. Our results indicate that while the incidence of clinical disease has continued to decline, cases occurred at any time of year. In contrast to previous surveys, affected farms were the same size as unaffected ones and there was no correlation between disease risk and the presence of deer on the land. Disease severity and mortality rates were increased because many infections were advanced by the time they were detected and treated. While the precise reasons for the decline in the incidence of redwater are unknown, a reversal of the trend could be devastating, as vigilance among farmers and veterinarians is flagging and the national herd is losing its protective immunity to disease.

